# Psychological Predictors of COVID-19 Prevention Behavior in Hungarian Women Across Different Generations

**DOI:** 10.3389/fpsyg.2021.596543

**Published:** 2021-01-26

**Authors:** Eszter Eniko Marschalko, Ibolya Kotta, Kinga Kalcza-Janosi, Kinga Szabo, Susana Jancso-Farcas

**Affiliations:** Department of Applied Psychology, Babes-Bolyai University, Cluj-Napoca, Romania

**Keywords:** COVID-19 prevention behavior, risk perception, psychological flexibility, negative automatic thoughts, health anxiety, generational differences, women

## Abstract

**Background:**

Age related differences were found in prevention behavior, showing that older individuals tend to be the most proactive. The aim of the study was the identification of psychological predictors on COVID-19 prevention behavior in women, across four generations. In addition, the predictive role of the psychological variables was explored through the lens of negative and positive information processing perspective on total and domain-specific COVID-19 prevention behavior.

**Methods:**

A cross-sectional research was conducted. The sample included 834 Hungarian speaking women. The assessed variables were: COVID-19 knowledge, risk perception, COVID-19 health anxiety, negative automatic thoughts, psychological flexibility, and four domains of COVID-19 prevention behavior (social distancing, general hygiene, information seeking, health behavior). A three-level hierarchical linear regression analysis was conducted to investigate the predictors of preventive behavior in each generation.

**Results:**

A diversity across generations was found. In case of baby boomer generation, the final model explained 32.4% of the variance for total prevention behavior [*F*(14,215) = 8.847, *p* < 0.001], and only perceived risk made a significant contribution. For Gen X the final model accounted for 21.1% of variance of total prevention behavior [*F*(14,341) = 7.788, *p* < 0.001], marital status, perceived risk, COVID-19 health anxiety, and negative automatic thoughts made significant contributions. In case of Gen Y the final model accounted for 6.2% of variance on total prevention behavior [*F*(14,147) = 1.761, *p* = 0.05], only perceived risk had a contribution to the final model. For Gen Z the final model accounted for 23.4% of variance on total preventive behavior [*F*(13,71) = 2.979, *p* = 0.002], and only psychological flexibility made a contribution to the model. The results on the distinct domains of COVID-19 prevention behavior emphasized details in the dissimilarity among generations.

**Conclusion:**

The role of generational identity on COVID-19 prevention behavior is relevant. The coexistence of negative and positive information processing may have its beneficial role in certain areas of prevention.

## Introduction

On the 11th of March 2020, the World Health Organization (WHO) officially declared the onset of a worldwide COVID-19 pandemic. Disease prevention behavior is essential in an emergency situation like this and individual action matters. The outbreak and rapid dissemination (Li et al., 2020) of the SARS-Cov-2 virus which caused the global spread of COVID-19 infections (World Health Organization, 2020a), ubiquitously influenced many governments to impose quarantine rules. During a disease outbreak, personal responsibility and also the degree of individual precautionary and preventive behavior can stop/delay unwanted consequences.

*Prevention behavior* is a list of actions linked to general hygiene, social distancing, healthy lifestyle, and can include any activity undertaken by a person who believes himself to be healthy for the purpose of preventing or detecting illness in an incipient state (Kasl and Cobb, 1966). World Health Organization (2019, 2020a) instructions in this pandemic were repeated and globally mediatized especially in relation to social distancing and hygienic guidelines. Furthermore, the majority of epidemiological studies from the literature assessed preventive behavior through relevant unicomponential aspects, which addressed avoiding individual infection as a way of preventing virus transmission (Tang and Wong, 2003; Choi and Kim, 2016; Jang et al., 2020; Li et al., 2020). The literature is sparse in multicomponential approach on COVID-19 prevention behavior. Social distancing (Abdelrahman, 2020; Iwaya et al., 2020) and hygiene (Li et al., 2020) were prioritized, but other important prevention domains like information seeking or health behavior/healthy lifestyle were less analyzed during this ongoing pandemic. There are findings that pinpoint the consequences of the pandemic on personal health behavior (Arora and Grey, 2020; Carroll et al., 2020), but less is known about the psychological determinants of this phenomenon. Misinformation was identified as a causal factor that hinders preventive behavior (Seitz, 2020), however, there is insufficient knowledge on psychological factors that play a role in active information-seeking behavior.

*Demographic variables* were often identified as holding a significant role in prevention behavior. During epidemiological events, compliance rates to precautionary measures are not always strong in the beginning. Psychological factors were often associated with demographic variables. The former are especially relevant because they have the potential to change.

*Age and gender* seem to have a significant positive role in influencing health risk assessments and behavioral adjustments (Krewski et al., 2006; Ibuka et al., 2010). The role of women in prevention behavior was highlighted (Ibuka et al., 2010), and especially older ones are more compliant to prevention guidelines (Clements, 2020; Dryhurst et al., 2020). Older individuals, like baby boomers respect more prevention guidelines, yet millennials are less engaged in prevention behaviors (Cherry and Morin, 2020).

*Marital status* plays a significant role in health behavior (Hilz and Wagner, 2018). The lack of a spouse increases the likelihood of health risk behaviors (Umberson, 1992; Norcross et al., 1996; Schone and Weinich, 1998; Kim et al., 2018).

*Education* can have a protective role, because it serves knowledge acquisition and its application (Krewski et al., 2006; Ibuka et al., 2010; Singh et al., 2020). People need time to decide what is potentially threatening to their well-being and health.

*Psychological factors* have an essential role in adherence to health maintenance guidelines. Epidemiological events can initiate negative assessments, which play a role in individual adaptation. The possible negative outcomes, threats, damages which can take place in a pandemic can trigger negative information-seeking and processing. Baumeister et al. (2001) highlighted the power of bad events over the good ones in human information processing and accommodation, as this probably serves a very important adaptive role.

*Knowledge about COVID-19* can support a more adjusted prevention behavior (e.g., Olapegba et al., 2020; Singh et al., 2020; Zegarra-Valdivia et al., 2020; Zhong et al., 2020). Relevant knowledge on a disease comprises etiology, symptoms, parameters of transmission, and treatment options. In case of the Ebola virus, the poor public comprehension of the malady contributed to its escalation (Ilesanmi and Alele, 2016). This point of view is upheld by various research, which evidence how the degree of information legitimately influences the control of transmission (Smith, 2006; Janjua et al., 2007; Ilesanmi and Alele, 2016; Zegarra-Valdivia et al., 2020). Olapegba et al. (2020) revealed that overall COVID-19 knowledge contributed to make feasible the control of the disease. A recent study found that in what concerns knowledge of COVID-19, baby boomers had significantly higher knowledge compared to members of Gen X, millennials, and Gen Z members (Clements, 2020). The rate of correct answers to COVID-19 related knowledge was influenced by age, educational level, type of occupation and household size (Singh et al., 2020). Knowledge about COVID-19 can be a critical variable that relates to positive attitudes toward preventive practices, but, on the other hand, knowledge was found insufficient in health behavior change and disease management (Duke et al., 2009; McAndrew et al., 2012). Moreover, World Health Organization (2020b) points out the gap between knowledge and practice in prevention. Accessing information and sometimes even knowledge about an illness is not enough for prevention.

*Risk perception*, for example, was revealed as a facilitator of prevention behavior (Dryhurst et al., 2020). Sheeran et al. (2014) meta-analysis showed that when interventions successfully change risk perception, then health behavior can change. Health related risk perception can be interpreted as one’s susceptibility to get an illness or a disease, and plays a significant role in prevention behavior (Ferrer and Klein, 2015). Former studies revealed that COVID-19 risk perception was positively associated with higher rates of precautionary actions (Karout et al., 2020). Gender related differences were found in risk perception and prevention behavior, as women tend to engage proactively, they perceive the risk of the disease more profoundly and even look into disease information more often (Ibuka et al., 2010). Age influences the subjective perceptions of risk associated with diseases and facilitates preventive behavior (Krewski et al., 2006). Significant differences in disease risk perception was revealed between generations and between genders (Tandi et al., 2018; Morgan et al., 2019; Mulia, 2019). Women in general and older groups reported greater concerns about health risk.

*COVID-19 health anxiety* was identified as a mental health consequence of the pandemic (Tyrer, 2020), and by definition can be identified as a constant fear of having or getting the disease. The fear of disease is a significant predictor of prevention behavior and it can change health related intentions, attitudes, and behaviors (Tannenbaum et al., 2015). It contributes to a misinterpretation (Salkovskis et al., 2002) of the communicated health-related information pertaining to COVID-19 management. A negative bias is present in information processing. Excessive media coverage of disease increases attentional focus on threat cues and heightens worry about the possibility of contamination (Taylor et al., 2000). Gender has been identified as a risk factor for COVID-19 anxiety, women being more exposed to this reaction (Özdin and Özdin, 2020). Higher fear of COVID-19 moderately predicted healthy behavior change, whilst perceived risk and symptoms of anxiety were found to have a significant, but small-to-moderate positive correlation with behavior change during the pandemic (Harper et al., 2020). According to the American Psychiatric Association (2017), when considering health-related fears prior to COVID-19 pandemic, baby boomers were less anxious than millennials about their health. Higher levels of health anxiety were found among younger adults (age 18–30 years) compared to their older counterparts (age 60–90 years), both as to illness likelihood and negative consequences (Salkovskis et al., 2002; Gerolimatos and Edelstein, 2012).

*Negative automatic thoughts* refer to negatively framed interpretations of what we think is going to happen to us (Beck, 1995). This negative repetitive thinking about causes and possible consequences increases negative thoughts about oneself, the future or the world, which can impede proper accommodation to required changes in health behavior to control the spread of COVID-19. The role of automatic negative thoughts was formerly analyzed in the context of a SARS epidemic. Khee et al. (2004) showed that overcoming the automatic negative thoughts through supportive therapy was helpful in coping with the challenges of the SARS, for healthcare providers, during the outbreak of the disease. These dysfunctional negative thoughts can make unbearable certain situations and can impede adjustment to change, unless they are modified. Cognitive theory posits that in stressful life circumstances automatic negative thoughts favor the development of depression (Beck, 1995). COVID-19 caused a global systematic shift (Chakraborty and Maity, 2020), social distancing and prevention behavior changed jobs, education, the daily routine of individuals. The change itself can be stressful, whereas routines and rituals in general have a beneficial impact on anxiety and stress (Hobson et al., 2017). Studies revealed that women in general seem to be more exposed to depression and anxiety disorders (Bahrami and Yousefi, 2011; Albert, 2015) and this justifies the assessment of the role of negative automatic thoughts that have been proved to hold an important role in psychological illness vs. health, which can influence preventive behavior. Several studies showed that depression is more prevalent in women of all ages, from adolescence to elderhood (Girgus et al., 2017), but little is known about negative automatic thoughts related to the quality of COVID-19 prevention behavior.

*Psychological flexibility* in contrast with the above psychological variables (perceived risk, COVID-19 health anxiety, negative automatic thoughts), is linked to a more positive reframing by promoting a growth perspective in information processing. It encourages cognitive reinterpretations, and summarizes five domains of reframing: positive perception of change, characterization of the self as flexible, characterization of the self as open and innovative, perception of reality as dynamic, changing and a perception of reality as multifaceted (Maor et al., 2014). Optimal levels of psychological flexibility add positive value to changing circumstances, makes adaptation to these conditions more accessible and brings about the possibility for openness. People with psychological flexibility can shift their focus from one life area to another, which helps them be more satisfied and balanced. This psychological factor characterized by reframing is nurturing acceptance of negative life events and can have an important role also in reducing the chance for pathological avoidance of undesired events, making the adjustment more resilient (Kashdan and Rottenberg, 2010; Dawson and Golijani-Moghaddam, 2020). Psychological flexibility can be a significant predictor of preventive health behavior, like influenza vaccine uptake (Cheung and Mak, 2016). It was identified as holding a significant role in resilient coping during the COVID-19 lockdown and in supporting mental health (Pakenham et al., 2020; Smith et al., 2020). Interventions in psychological flexibility gained empirical support in addressing mental health problems caused by the COVID-19 pandemic (Landi et al., 2020; Polizzi et al., 2020). Better personal adjustment strategies were facilitated by psychological flexibility and lowered the chance of mental health problems in COVID-19 pandemic (Dawson and Golijani-Moghaddam, 2020; Puolakanaho et al., 2020). It is considered a key element of psychological resilience, because if one is able to think flexibly, then he or she tends to be more resilient facing a stressful situation and can accept challenging situations and control their own behavior in case something unexpected occurs (Haglund et al., 2007).

*The role of bad and good information processing* in health was the theoretical frame that inspired the current study. “Bad is stronger than good,” as a general principle, was suggested as definitory for human health and health related behavior (Baumeister et al., 2001). Bad or negative information is processed more profoundly, has a greater impact among individuals and it was considered highly adaptive in an evolutionary approach. Epidemiological events can be considered negative, being associated with physical and everyday life threats and negative emotions. Based on this principle, negative information processing related variables (e.g., risk perception, COVID-19 health anxiety, negative automatic thoughts), can influence preventive behavior. Contrary to this perspective, optimism, and personal control also has an important role in health and health sustaining behavior (Taylor et al., 2000). Positive information related psychological variables, which involve beneficial self-related resources (e.g., psychological flexibility) are linked to a more positive reframing and also can have a role in COVID-19 preventive behavior.

### Aim of the Study

The majority of formerly presented studies pinpointed that women (Ibuka et al., 2010), and especially older ones are more compliant to prevention guidelines (Clements, 2020; Dryhurst et al., 2020). In different studies, perceived risk, health anxiety, negative automatic thoughts were highlighted as more prevalent in female participants (Bahrami and Yousefi, 2011; Albert, 2015; Girgus et al., 2017). This research was conducted on a multigenerational sample of women, considering that their role was proven to be that of facilitators of disease prevention and yet little is known about the age or generational attributes they share.

The main aim of the study was to explore the predictive role of the above-mentioned psychological factors (perceived risk, COVID-19 health anxiety, negative automatic thoughts, and psychological flexibility) on the SARS-CoV-2 prevention behavior in general (considering all areas) and in a domain-specific way. The assumption of diversity in generational identity could lead to a fresh perspective on the topic. Based on the literature considered above, it was assumed that risk perception (Brug et al., 2004; Ibuka et al., 2010; Commodari, 2017) and COVID-19 health anxiety (Salkovskis et al., 2002) are significant positive predictors on COVID-19 preventive behavior. In case of psychological flexibility and negative automatic thoughts these analyses are exploratory. In addition, the predictive role of the aforementioned psychological predictors were explored through the lens of negative and positive information processing perspective on different domains of COVID-19 prevention behavior (social distancing, general hygiene, information seeking, and health behavior). Literature reviews highlighted the function of negative and positive information processing variables, as well, in personal accommodation and health (Taylor et al., 2000; Baumeister et al., 2001).

## Measurement and Methods

### Participants

The sample included 834 Hungarian speaking women. Considering their residence 80% were from Hungary, 15% from Romania, and 5% from other countries (Sweden, Germany, Ireland, and United Kingdom). All participants’ first language was Hungarian. The sociodemographic characteristics of participants are presented in Table 1.

**TABLE 1:** Baseline characteristics of the participants (*N* = 834).

	**Baby boomer (*n* = 230)**	**Gen X (*n* = 356)**	**Gen Y (*n* = 163)**	**Gen Z (*n* = 85)**	***F*/*X*^2^ (df)**
Age	62.30 ± 4.66	48.12 ± 4.29	34.73 ± 4.47	20.51 ± 1.94)	2538.81 (3,830)**
Education (*n*, *%*)					51.95 (12)**
8th grade or less	1 (0.4%)	2 (0.6%)	–	2 (2.4%)	
Baccalaureate	60 (26.1%)	108 (30.3%)	28 (17.2%)	42 (49.4%)	
College, university	149 (64.8%)	210 (59.0%)	101 (62.0%)	39 (45.9%)	
Master’s degree	13 (5.7%)	23 (6.5%)	26 (16.0%)	1 (1.2%)	
Doctorate	7 (3.0%)	13 (3.7%)	8 (4.9%)	1 (1.2%)	
Residency (*n*, *%*)					34.25 (6)**
Capital city	57 (24.8%)	88 (24.7%)	38 (23.3%)	3 (3.5%)	
City	136 (59.1%)	186 (52.2%)	83 (50.9%)	54 (63.5%)	
Village	37 (16.1%)	82 (23.0%)	42 (25.8%)	28 (32.9%)	
Marital status (*n*, %)					103.34 (9)**
Single	25 (10.9%)	48 (13.5%)	34 (20.9%)	36 (42.4%)	
Relationship or married	135 (58.7%)	248 (69.7%)	116 (71.2%)	47 (55.3%)	
Divorced	51 (22.2%)	57 (16.0%)	11 (6.7%)	–	
Other	19 (8.3%)	3 (0.8%)	2 (1.2%)	2 (2.4%)	
Number of children	1.92 ± 1.02	1.69 ± 1.19	0.88 ± 1.13	0.02 ± 0.21	85.08 (3,830)**
Household size	1.96 ± 1.10	3.13 ± 2.10	2.91 ± 1.31	3.99 ± 1.08	39.50 (3,830)**
Infected acquaintances (*n of yes*, %)	31 (13.5%)	47 (13.2%)	24 (14.7%)	8 (9.4%)	1.50 (3)
Knowledge	6.16 ± 0.77	6.19 ± 0.73	6.41 ± 0.68	6.35 ± 0.76	4.80 (3,830)**
Perceived risk	20.66 ± 6.31	20.81 ± 5.41	20.75 ± 5.19	20.57 ± 4.37	0.07 (3,312.68)^1^
COVID-19 health anxiety	3.80 ± 2.37	4.23 ± 2.56	4.64 ± 2.64	4.43 ± 2.10	4.01 (3,829)**
Negative automatic thoughts	22.16 ± 7.36	24.51 ± 9.97	27.23 ± 10.81	31.21 ± 11.30	20.98 (3,286.09)^1^**
Psychological flexibility	25.19 ± 3.04	25.00 ± 2.97	24.64 ± 3.10	23.56 ± 3.31	6.50 (3,830)**
Preventive behavior	12.18 ± 1.87	11.61 ± 1.72	11.61 ± 1.62	11.67 ± 1.60	5.803 (3,830)**
Social distancing	3.34 ± 0.59	3.25 ± 0.55	3.42 ± 0.43	3.57 ± 0.47	11.708 (3,309.99)^1^**
Health behavior	3.29 ± 0.56	3.15 ± 0.65	3.14 ± 0.63	3.16 ± 0.65	3.26 (3,295.77)^1^*
General hygiene	2.62 ± 0.82	2.37 ± 0.74	2.37 ± 0.76	2.36 ± 0.73	5.72 (3,298.27)^1^**
Information seeking	2.92 ± 0.92	2.85 ± 0.87	2.70 ± 0.87	2.58 ± 0.83	4.39 (3,830)**

*p < 0.05, **p < 0.01. ^1^Welch ANOVA.

### Measurements

#### Socio-demographic Information and COVID-19 Related Variables

The demographic and COVID-19 background factors included the following: age, gender, education, residency, number of children, the household size, acquaintances infected with COVID-19 (yes vs. no).

*Knowledge of COVID-19* (adapted from Cheng and Ng, 2006) was measured by seven items rated by true or false (scored “1,” and “0,”respectively). Three of the items are correct statements about COVID-19 (“Fever is a major symptom of COVID-19”; “A vaccine is not yet available for preventing COVID-19”; and “The incubation period of COVID-19 can be 14 days”). The other four are incorrect statements (“Transmission of COVID-19 is mainly by air/airborne transmission”; “COVID-19 is caused by a kind of bacteria called streptococcus”; “COVID-19 can be transmitted through handling dollar notes [bills] and coins”; “”Children are most vulnerable to COVID-19”). Participants chose one of the two options (True, False) for each of the items. Correct answers received a score point and incorrect answers received no score points. The overall knowledge score, ranging from 0 to 7, sums up the score points of all items. A higher score indicates more accurate knowledge about COVID-19.

#### COVID-19 Prevention Behavior

This scale was developed for the current study. The instrument is not a diagnostic scale; it measures only preventive behavioral tendencies. The initial questionnaire includes 20 items, inspired by previous published research on pandemics and epidemics (e.g., Cheng and Ng, 2006) and the WHO prevention recommendations (World Health Organization, 2019). The initial scale covered five dimensions of prevention behavior: social distancing (sample item, e.g., “I avoid going out to eat.”), general hygiene (sample item, e.g., “I wear gloves.”), hand hygiene (sample item, e.g., “I wash hands with soap before eating.”), information seeking (sample item, e.g., “I seek information/advice from healthcare workers.”), and health behavior (e.g., healthy eating and sport) (sample item, e.g., “I get an adequate amount of sleep and rest”). Based on preliminary results (factor structure and correlations between subscales), the Hand Hygiene (hand washing) subscale in this study was not used, because the questions were covered by the general hygiene subscale. The items were rated on a 4-point Likert scale from 1 (very unlikely) to 4 (very likely) (sample item, e.g., “I clean the house with disinfectant or a diluted bleach.”). Total score was computed from subscale means (range 4–16). The final scale contains 18 items and has good total reliability (Cronbach’s α = 0.79). The Cronbach alpha values for the subscales were as follows: social distancing, α = 0.70, general hygiene α = 0.78, information seeking α = 0.61, and health behavior α = 0.552. The last subscale (health behavior) comprised only three items for three actions (sleep quality, sport, and healthy diet), which can justify the lower reliability value. Because the results were not used for specific diagnostics, the last subscale was included in the planned analysis, considering the moderate value of reliability (Hinton et al., 2004).

#### Perceived Risk

The scale included seven items regarding risk perception of the new coronavirus inspired by previous researchers investigating other outbreaks (e.g., Brug et al., 2004; Ibuka et al., 2010; Commodari, 2017). Among the seven items about risk perception (Cronbach’s α = 0.81), two items (1, 3) investigated the likelihood of contracting coronavirus, three items (2, 4, and 5) dealt with the general severity of the disease and two items (6, 7) were about the perceived susceptibility to disease. The seven items were rated on a 5-point scale ranging from 1 to 5 (not agree at all, totally agree) (sample item, e.g., “There is a high probability that I will encounter somebody infected with coronavirus (COVID-19).” Higher scores suggest perceptions of greater risk.

#### Psychological Flexibility

The scale comprised of 20 items and were developed by Maor et al. (2014) and measured five dimensions of psychological flexibility: positive perception of change, characterization of the self as flexible, characterization of the self as open and innovative, perception of reality as dynamic and changing and a perception of reality as multifaceted. All items were rated on a 6-point scale ranging from 1 (not agree at all) to 6 (totally agree) (sample item, e.g., “I often find change to be a challenge.”). Mean subscale scores were summed to obtain the total score of the scale (range 5–30). Higher scores suggest greater levels of psychological flexibility. The total score of the scale in the current study has an excellent reliability level (Cronbach’s α = 0.91).

#### Negative Automatic Thoughts

The 15-item Automatic Thoughts Questionnaire (ATQ; Netemeyer et al., 2002) is a shortened version of Hollon and Kendall’s (1980) scale. It contains 15 statements describing negative/dysfunctional self-related automatic thoughts. Subjects are asked to rate the frequency of occurrence of these thoughts on a 5-point Likert scale ranging from 1 (never) to 5 (almost always) (sample item, e.g., “I can’t stand this anymore.”). Higher ATQ scores indicate high presence of dysfunctional/irrational automatic thoughts. Netemeyer et al. (2002) reported reliability estimates of 0.96 for the 15-item ATQ. Coefficient alpha estimates of internal reliability for the present study was 0.94, signaling we have a psychometrically excellent measure.

*COVID-19 health anxiety* was measured by six items from Health Anxiety Inventory – short version (Salkovskis et al., 2002). The development and validation of the original inventory, which inspired us, was based on the assumption of the authors concerning possible misinterpretation of ambiguous health-related information (external and internal stimuli, a body centered perspective/external and internal bodily stimuli). The following items: 1, 4, 6, 7, 9, 11 were rewritten with reference to COVID-19 pandemic. In the inventory we investigated the presence of fear and worry about COVID-19 illness was investigated: (1), psychological reactions to bodily sensations (4, 7), concerns about death (6), preoccupation, interference and bodily awareness (9), and deliberate action determined by bodily sensations (11). Higher score suggests higher COVID-19 anxiety. Items were scored on a Likert-type scale from 0 to 3 [sample item, e.g., “I usually feel at very low/fairly/moderate/high risk for developing the infection caused by the new Coronavirus (COVID-19).”]. The Cronbach’s α of the inventory was 0.77.

### Procedure

This was a cross-sectional study. The protocol of the study was approved by the Ethics Committee of the University of Babeş-Bolyai (RO). A complete description of the study was provided to all subjects and they all gave their consent to participate in this investigation. The online data collection was initiated on 15th of April 2020 and closed on 15th of May 2020. An online questionnaire package was developed on Google Forms, with a consent form appended to it. The link of the questionnaire was sent through social media to the contacts of the investigators. Data was collected using self-reported questionnaires. The participation was voluntary. Participants older than 18 years of age, Hungarian speaking and willing to give informed consent were included in the study. Data was collected from Hungary, Romania, Sweden, Germany, Ireland, and the United Kingdom. A Snowball sampling technique was used. For data analysis, SPSS (Statistical Package for the Social Sciences version 23.0) software was used.

### Data Screening

The first set of tests included the screening data analysis with steps established by Field (2009) and Tabachnick and Fidell (2013). Based on the missing values analysis there were no variables with 5% or more missing values. Univariate outlier analyses were conducted using *z*-score analysis among the major continuous variables. A total of 95% of cases had an absolute value less than 1.96 and none of the cases had a value greater than 3.29. Normality distribution assumptions were checked applying exploratory data analysis. Due to the large sample size (*N* = 834), the significance test of skew and kurtosis was not applied. Based on visual analysis of histograms and Q–Q plot of the data, the sample has a mostly normal distribution.

All the data were presented as mean (*M*) and standard deviation (SD) for continuous variables and frequencies/percentages for categorical variables. A *p*-value of less than 0.05 was considered statistically significant. Differences between generational groups (baby boomers, Gen X, Gen Y, Gen Z) were tested at the baseline with analysis of variance (one-way ANOVA) for continuous outcomes and Pearson’s Chi-square for categorical outcomes.

Initially, the homogeneity of variances was checked with the Levene statistics and whenever the assumption of homogeneity was not met, the robust Welch F-test of equality of means was interpreted. For equal variances pairwise comparisons the Scheffé’s procedure and for unequal variances Games-Howell *post hoc* analysis was performed. These are the most general and flexible, but also the most conservative tests. Cohen’s *f* effect sizes were computed and interpreted via the Webpower package developed by Zhang and Yuan (2018).

A three-level hierarchical linear regression analysis was conducted to investigate the predictors of preventive behavior separately, for each generational group.

Data screening analyses were conducted to ensure no violation of the assumptions of normality of residuals, linearity, multicollinearity, and homoscedasticity based on Tabachnick and Fidell’s (2013) guidelines. Durbin Watson statistic was used to test the autocorrelation in the residuals and linearity. No problems of multicollinearity between the variables used in the regression were identified, all bivariate correlations were found to be less than 0.9 and the variance inflation factor <3.0. The variables for the regression models were selected based on theory and statistical analysis.

The first step addressed socio-demographic variables of interest (education, residency, marital status, number of children, household size) and a COVID-19 related variable (infected acquaintances). From this point on, for the sake of brevity, infected acquaintances will be referred to as socio-demographic variable. The second step added Knowledge about COVID-19. The first two models were used for the purpose of statistical control. In the third step, psychological factors (risk perception, COVID-19 health anxiety, automatic thoughts, psychological flexibility) were added to identify any additional variance that may predict preventive behavior.

Table 2 shows the *R*^2^ and Δ*R*^2^ after entering variables at each step, as well as the unstandardized regression coefficients (*B*), standard errors (SE), standardized regression coefficients (β) and *t*-values from the final model.

**TABLE 2:** Hierarchical regression results for preventive behavior.

	**1 Baby boomer generation**									**2 Generation X**								
	**Model 1**			**Model 2**			**Model 3**			**Model 1**			**Model 2**			**Model 3**		
	***B***	**β**	**SE**	***B***	**β**	**SE**	***B***	**β**	**SE**	***B***	**β**	**SE**	***B***	**β**	**SE**	***B***	**β**	**SE**
(Constant)	11.69**		0.69	7.84**		1.14	5.96**		1.40	11.29**		0.43	10.08**		0, 85	8.08**		1.18
Education level^*a*^	0.26	0.09	0.19	0.17	0.06	0.19	0.20	0.07	0.16	0.17	0.07	0.13	0.16	0.07	0.13	–0.03	–0.01	0.12
Residency																		
City^*b*^	0.08	0.02	0.31	0.22	0.06	0.30	0.04	0.01	0.26	0.17	0.04	0.23	0.17	0.04	0.23	0.15	0.04	0.21
Village^*c*^	0.37	0.07	0.42	0.45	0.09	0.41	–0.06	–0.01	0.35	–0.24	–0.06	0.23	–0.26	–0.06	0.23	–0.09	–0.02	0.21
Marital Status																		
Single^*d*^	–0.10	–0.02	0.45	0.02	0.00	0.43	–0.11	–0.02	0.38	–0.42	–0.08	0.29	–0.44	–0.09	0.29	–0.43	–0.09	0.26
Divorced^*e*^	–0.07	–0.02	0.35	0.01	0.00	0.33	–0.04	–0.01	0.28	−0.66*	−0.14*	0.26	−0.67*	−0.14*	0.26	−0.57*	−0.12*	0.23
Other^*f*^	–0.81	–0.12	0.47	–0.61	–0.09	0.45	–0.23	–0.03	0.39	1.04	0.05	1.01	0.93	0.05	1.01	0.30	0.02	0.92
Number of children^*g*^	–0.05	–0.03	0.13	–0.07	–0.04	0.12	–0.04	–0.02	0.11	0.01	0.01	0.09	0.01	0.01	0.09	0.01	0.01	0.08
Household size^*h*^	–0.11	–0.07	0.13	–0.17	–0.10	0.12	–0.09	–0.05	0.11	–0.02	–0.02	0.05	–0.02	–0.03	0.05	–0.02	–0.03	0.04
Infected aquintances	0.41	0.08	0.37	0.57	0.10	0.36	0.14	0.03	0.31	0.41	0.08	0.27	0.40	0.08	0.27	0.01	0.01	0.24
Knowledge^*j*^				0.66**	0.28**	0.16	0.24	0.10	0.14				0.20	0.09	0.12	0.11	0.05	0.11
Perceived risk^*k*^							0.15**	0.50**	0.02							0.10	0.30**	0.02
COVID-19 health anxiety^*l*^							0.07	0.09	0.06							0.15	0.22**	0.04
Negative automatic thoughts^*m*^							0.00	–0.01	0.02							–0.03	−0.19**	0.01
Psychological flexibility^*n*^							0.05	0.08	0.04							0.05	0.08	0.03
*R*^2^	0.03			0.10			0.37			0.04			0.05			0.24		
Adj. *R*^2^	–0.01			0.06*			0.32**			0.01			0.02			0.21**		
Δ*R*^2^				0.07**			0.26**						0.01			0.20**		
	**3 Generation Y**										**4 Generation Z**							
(Constant)	12.07**		0.73	12.09**		1.45	9.67**		1.86	11.49**		1.09	11.13**		1.84	7.76**		1.95
Education level^*a*^	–0.03	–0.01	0.19	–0.03	–0.01	0.19	–0.16	–0.07	0.18	0.07	0.03	0.32	0.07	0.03	0.32	–0.04	–0.02	0.29
Residency																		
City^*b*^	0.06	0.01	0.34	0.06	0.01	0.34	–0.15	–0.04	0.33	1.84	0.21	1.09	1.85	0.21	1.10	1.16	0.13	1.01
Village^*c*^	0.19	0.05	0.33	0.19	0.05	0.33	0.11	0.03	0.31	0.61	0.18	0.36	0.61	0.18	0.37	0.49	0.14	0.34
Marital status																		
Single^*d*^	–0.01	0.00	0.35	–0.01	0.00	0.36	–0.14	–0.04	0.34	0.08	0.02	0.35	0.08	0.03	0.35	–0.14	–0.04	0.33
Divorced^*e*^	–0.55	–0.09	0.62	–0.55	–0.09	0.63	–0.39	–0.06	0.60									
Other^*f*^	–1.34	–0.09	1.26	–1.34	–0.09	1.28	–1.43	–0.10	1.22	−2.39*	−0.23*	1.13	−2.37*	−0.22*	1.14	–1.82	–0.17	1.05
Number of children^*g*^	0.21	0.15	0.19	0.21	0.15	0.19	0.14	0.10	0.19	–0.10	–0.01	0.87	–0.12	–0.02	0.87	–0.12	–0.02	0.80
Household size^*h*^	–0.19	–0.16	0.17	–0.19	–0.16	0.17	–0.14	–0.11	0.16	–0.04	–0.03	0.17	–0.03	–0.02	0.17	0.01	0.00	0.16
Infected acquintances^*i*^	0.12	0.03	0.38	0.12	0.03	0.38	0.03	0.01	0.36	–0.88	–0.16	0.63	–0.88	–0.16	0.63	–0.65	–0.12	0.58
Knowledge^*j*^				0.00	0.00	0.20	–0.03	–0.01	0.19				0.06	0.03	0.23	–0.18	–0.08	0.21
Perceived risk^*k*^							0.08*	0.26*	0.03							0.05	0.15	0.05
COVID-19 health anxiety^*l*^							0.08	0.12	0.06							0.01	0.01	0.09
Negative automatic thoughts^*m*^							–0.02	–0.12	0.01							–0.01	–0.10	0.01
Psychological flexibility^*n*^							0.06	0.11	0.04							0.18**	0.38**	0.05
*R*^2^	0.02			0.02			0.14			0.16			0.16			0.35		
Adj. *R*^2^	–0.04			–0.04			0.06*			0.07			0.06			0.23*		
Δ*R*^2^				0.00			0.12*						0.01			0.19*		

N = 834, *n*1 = 230, *n*2 = 356, *n*3 = 163, *n*4 = 85. We examined the impact of sociodemographic factors, knowledge about COVID-19 and psychological factors, such as risk perception, health anxiety, automatic thoughts, psychological flexibility – on preventive behavior. In Model 1, we entered sociodemographic factors: education level, residence, marital status, number of children, household size, as control variables to predict preventive behavior. In Model 2, we entered knowledge about COVID-19 as a predictor. In Model 3 psychological factors such as risk perception, health anxiety, automatic thoughts, psychological flexibility were used to predict preventive behavior. *p < 0.05, **p < 0.01. ^*a*^1 = 8th grade or less, 2 = baccalaureate, 3 = college, university, 4 = master’s, 5 = doctorate; ^*b*^1 = city, 0 = capital city, village; ^*c*^1 = village, 0 = city, capital city; ^*d*^1 = single, 0 = living in a relationship, married, divorced, other; ^*e*^1 = divorced, 0 = single, living in a relationship, married, other; ^*f*^1 = other, 0 = single, living in a relationship, married, divorced; ^*g*^ = number of children, ^*h*^ = household size, ^*i*^1 = yes, 0 = no; ^*j*^1 = true, 0 = false; ^*k*^1 = not agree at all, 2 = not agree, 3 = neither agree nor disagree, 4 = agree, 5 = totally agree; ^*l*^0 = not agree at all, 1 = not agree, 2 = agree, 3 = totally agree; ^*m*^1 = not at all, 2 = sometimes, 3 = moderately often, 4 = often, 5 = all the time; ^*n*^1 = not agree at all, 2 = not agree, 3 = neither agree nor disagree, 4 = somewhat agree, 5 = agree, 6 = totally.

## Results

### *A priori* Power Analysis

*A priori* power analysis via G^∗^Power3 (Faul et al., 2007) for hierarchical linear regression based on type I error with a *p*-value of 0.05 and statistical power of 0.80 with four tested predictors (total number of predictors 11) showed that for a medium effect size (*f*^2^ = 0.15) the required sample size is *n* = 85, while for a small effect size (*f*^2^ = 0.02) the required sample size is *n* = 602. Thus, the sample sizes of all generational groups (*n*_babyboomer_ = 85, *n*_Gen X_ = 356, *n*_Gen Y_ = 162, and *n*_Gen Z_ = 85) are suitable for detecting medium effect sizes.

### Generational Differences in Psychological Predictors

Generational differences were analyzed along the psychological predictors of preventive behavior (see Table 1 for one-way ANOVA F-values and significance).

#### Knowledge of COVID-19

A one-way analysis of variance showed that the difference between generations in knowledge of COVID-19 was significant with *Cohen’s f* = 0.13. *Post hoc* analyses using the Scheffé *post hoc* criterion for significance indicated that the knowledge of COVID-19 was significantly higher in Gen Y (*M* = 6.41, SD = 0.68) than in Gen X (*M* = 6.20, SD = 0.74), *p* = 0.026, 95% CI (0.02, 0.41), *Cohen’s f* = 0.11, and in baby boomers (*M* = 6.16, SD = 0.78), *p* = 0.013, 95% CI (0.04, 0.46), *Cohen’s f* = 0.11.

#### Perceived Risk

No generational differences were found for perceived risk (see Table 1).

#### COVID-19 Health Anxiety

A one-way analysis of variance showed that the difference between generations in COVID-19 health anxiety was significant with *Cohen’s f* = 0.12. *Post hoc* analyses using the Scheffé *post hoc* criterion for significance indicated that the COVID-19 health anxiety was significantly higher in Gen Y (*M* = 4.65, SD = 2.64) than in baby boomers (*M* = 3.80, SD = 2.38), *p* = 0.012, 95% CI (0.13, 1.56), *Cohen’s f* = 0.12.

#### Psychological Flexibility

A one-way analysis of variance showed that the difference between generations in psychological flexibility was significant with *Cohen’s f* = 0.15. *Post hoc* analyses using the Scheffé *post hoc* criterion for significance indicated that the psychological flexibility was significantly smaller in Gen Z (*M* = 23.57, SD = 3.31) than in Gen X (*M* = 25.00, SD = 2.98), *p* = 0.002, 95% CI (−2.47, −0.41), *Cohen’s f* = 0.13 and in baby boomer (*M* = 25.19, SD = 3.04), *p* = 0.001, 95% CI (−2.71, −0.53), *Cohen’s f* = 0.14.

#### Negative Automatic Thoughts

The assumption of the homogeneity of variances was not assumed (Levene statistics 13.52, *p* < 0.001). The robust Welch one-way ANOVA showed that the difference between generations in negative automatic thoughts was significant with *Cohen’s f* = 0.27. Games-Howell *post hoc* analysis revealed significant differences between all generations baby boomer (*M* = 22.16, SD = 7.36), Gen X (*M* = 24.52, SD = 9.97), Gen Y (*M* = 27.24, SD = 10.81), Gen Z (*M* = 31.21, SD = 11.30). All *p*-values were <.05 and *Cohen’s f* values from lowest 0.10 (between baby boomers and Gen X) to highest 0.25 (between baby boomers and Gen Z).

### Results for Baby Boomer Generation

The results of the hierarchical regression demonstrated that the sociodemographic variables independently accounted for a non-significant 3.4% of variance in preventive behavior scores. In Step 2, knowledge of COVID-19 reliably predicted preventive behavior and explained an additional 7% of the variance, Δ*R*^2^ = 0.070, *F*_change_(1,219) = 17.075, *p* < 0.001. The addition of psychological factors in Step 3 significantly added further 26.2% to the model’s predictive power after controlling for sociodemographic factors and knowledge of COVID-19, Δ*R*^2^ = 0.262, *F*_change_(4,215) = 22.203, *p* <. 001. The adjusted *R*^2^ values in Table 2 show increases in the variance explained by each successive model, with the final model explaining 32.4% of the variance for preventive behavior, *F*(14,215) = 8.847, *p* < 0.001. The total effect size of the regression for the baby boomers (*n* = 230) Cohen’s *f*^2^ = 0.41 was large. Only perceived risk (β = 0.504, *p* < 0.001) made a unique, statistically significant contribution to the final model predicting preventive behavior.

Considering the different subscales of preventive behavior as outcome variables with the same predictors at Step 1 (socio-demographic variables), 2 (knowledge of COVID-19), and 3 (psychological factors), data analysis revealed that the sociodemographic variables contributed non-significantly to the variance in the outcome variable (6.8, 2.6, 2.8, and 2.9% for social distancing, general hygiene, information seeking, and health behavior, respectively).

*For social distancing*, knowledge of COVID-19 was a significant predictor adding a significant 5.8% of variance, *F*_change_(1,219) = 14.596, *p* < 0.001 in social distancing scores and psychological factors added significant 17.8% of variance *F*_change_(4,215) = 13.771, *p* < 0.001. The final model accounted for 25.9% of variance of social distancing, *F*(14,215) = 6.717, *p* < 0.001, as indexed by adjusted *R*^2^ statistic. Only marital status (β = −0.156, *p* = 0.01) and perceived risk (β = 0.444, *p* < 0.001) made a unique statistically significant contribution to the final model predicting social distancing.

For *general hygiene*, the addition of knowledge of COVID-19 in the second block was significant, explaining 5% of variance *F*_change_ (1,219) = 11.797, *p* = 0.001, while psychological factors in the third block significantly added an extra 17.4% *F*_change_(4,215) = 12.422, *p* < 0.001, in general hygiene scores. As indexed by adjusted *R*^2^ statistic, the final model accounted for 20% of variance in general hygiene, *F* (14,215) = 5.091, *p* < 0.001. Only perceived risk (β = 0.447, *p* < 0.001) made a unique statistically significant contribution to the final model predicting general hygiene.

For *information seeking*, knowledge of COVID-19 in Step 2 added a significant 2.3% of variance to the model *F*_change_(1,219) = 5.422, *p* = 0.021, and psychological factors in Step 3 added another 28.2% of explained variance *F*_change_(4,215) = 22.757, *p* < 0.001. The final model accounted for 29.1% of variance in information seeking, *F*(14,215) = 7.699, *p* < 0.001. Along perceived risk (β = 0.337, *p* < 0.001), health anxiety (β = 0.208, *p* = 0.006) and automatic thoughts (β = 0.157, *p* = 0.009) also made a unique statistically significant contribution to the final model predicting information seeking.

For *health behavior*, knowledge of COVID-19 did not add significantly to the model in Step 2 *F*_change_(1,219) = 0.612, *p* = 0.435, while psychological factors added 10% to the model in Step 3 *F*_change_(4,215) = 6,165, *p* < 0.001. The final model explained 7.4% of variance in health behavior subscale, *F*(14,215) = 2.315, *p* = 0.006. Only automatic thoughts (β = −0.226, *p* < 0.001) and psychological flexibility (β = 0.144, *p* < 0.05) had a unique significant contribution to the model.

### Results for Generation X

At Step 1, sociodemographic variables contributed a non-significant 3.9% of variance. The addition of knowledge of COVID-19 at Step 2 was not significant, adding only 0.8% of variance, *F*_change_(1,345) = 2.729, *p* = 0.099. At Step 3, psychological factors added significant 19.5% of variance, *F*_change_(4,341) = 21.971, *p* < 0.001. As indexed by adjusted *R*^2^ statistic, the final model accounted for 21.1% of variance of preventive behavior, *F*(14,341) = 7.788, *p* < 0.001. The total effect size of the regression for Gen X (*n* = 356) Cohen’s *f*^2^ = 0.257 was medium. Divorced marital status (β = −0.122, *p* = 0.014), perceived risk (β = 0.309, *p* < 0.001), COVID-19 health anxiety (β = 0.225, *p* < 0.001), and automatic thoughts (β = −0.189, *p* < 0.001) made unique, statistically significant contributions to the final model, predicting preventive behavior.

For *social distancing*, socio-demographic variables added at Step 1 revealed a significant model accounting for only 5.6% in the variation of the outcome variable, *F*_change_(9,346) = 2.262, *p* = 0.018. At Step 2, knowledge of COVID-19 significantly added 2.1%, *F*_change_(1,345) = 7.902, *p* = 0.005, while psychological factors at Step 3 added a further 9.5% of variance, *F*_change_(4,341) = 9.735, *p* < 0.001. Adjusted *R*^2^ shows that the final model accounts for 13.7% of the variance in social distancing, *F*(14,341) = 5.036, *p* < 0.001. Marital status (single β = −0.113, *p* = 0.036; divorced β = −0.133, *p* = 0.010), knowledge of COVID-19 (β = 0.116, *p* = 0.022), perceived risk (β = 0.177, *p* = 0.003) and health anxiety (β = 0.194, *p* = 0.002) had a unique significant contribution to the final model predicting preventive behavior.

For *general hygiene*, socio-demographic variables at Step 1 resulted in an insignificant model, explaining only 2.2% of the outcome variable, *F*_change_(9,346) = 0.880, *p* = 0.543. Knowledge of COVID-19 added a significant 1.2%, *F*_change_(1,345) = 4.196, *p* = 0.041 at Step 2 and psychological factors accounted for further 12% of the model, *F*_change_(4,341) = 12.033, *p* < 0.001. The final model explained 12% of variance in general hygiene, *F*(14,341) = 4.420, *p* < 0.001. Education (β = −0.161, *p* = 0.002), perceived risk (β = 0.269, *p* < 0.001), health anxiety (β = 0.137, *p* = 0.029) and automatic thoughts (β = −0.163, *p* = 0.003) had a unique significant contribution to the final model.

For *information seeking*, socio-demographic variables resulted in a significant model, explaining 6.5% of variance in the outcome variable. At Step 2, knowledge of COVID-19 added a nonsignificant 0.1% to the model *F*_change_(1,345) = 0.538, *p* = 0.464, while psychological factors explained an additional 23.2% of variance, *F*_change_(4,341) = 28.212, *p* < 0.001. The final model explained 27% of the variance in information seeking, as indicated by the adjusted *R*^2^ value, *F*(14,341) = 10.373, *p* < 0.001. Residency (city β = −0.138, *p* = 0.018; village β = −0.125, *p* = 0.037), perceived risk (β = 0.379, *p* < 0.001), and health anxiety (β = 0.196, *p* = 0.001) had a significant unique contribution to the final model.

For *health behavior*, the introduction of sociodemographic variables at Step 1 resulted in an insignificant model, explaining 4.6% of variance in the outcome variable, *F*_change_(9,346) = 1.839, *p* = 0.060. The addition of knowledge of COVID-19 at Step 2 added a nonsignificant 0.4% to the model, *F*_change_(1,345) = 1.620, *p* = 0.204, while psychological factors significantly explained 15.3% of variance in health behavior, *F*_change_(4,341) = 16.387, *p* < 0.001. The final model explained 17.1% of the variance in health behavior, as indexed by adjusted *R*^2^, *F*(14,341) = 6.214, *p* < 0.001. Education (β = 0.113, *p* = 0.024), perceived risk (β = −0.145, *p* = 0.014), automatic thoughts (β = −0.236, *p* < 0.001) and psychological flexibility (β = 0.200, *p* < 0.001) all added a unique significant contribution to the final model.

### Results for Generation Y

Socio-demographic variables added at Step 1 revealed a non-significant model accounting for only 2.2% in the variation of preventive behavior. Knowledge of COVID-19 added 0% of variance at Step 2. The addition of psychological factors at the third block significantly added 12.2% of variance after controlling for sociodemographic variables and knowledge of COVID-19, *F*_change_(4,147) = 5.239, *p* = 0.001. The final model as a whole accounted for 6.2% of variance in preventive behavior, as indexed by adjusted *R*^2^ statistic, *F*(14,147) = 1.761, *p* = 0.05. The total effect size of the regression for Gen Y (*n* = 162) Cohen’s *f*^2^ = 0.142 was small. Only perceived risk (β = 0.256, *p* = 0.007) had a unique significant contribution to the final model predicting preventive behavior.

For *social distancing*, socio-demographic variables added at Step 1 resulted in a significant model, explaining 12.4% of variance in the outcome variable, *F*_change_(9,152) = 2.384, *p* = 0.015. At Step 2, the addition of knowledge of COVID-19 was insignificant, *F*_change_(1,151) = 1.902, *p* = 0.170. At Step 3, psychological factors added a significant 6.3% to the model, *F*_change_(4,147) = 2.880, *p* = 0.025. The final model explained 12.1% of the variance in social distancing, as indexed by adjusted *R*^2^ statistic, *F*(14,147) = 2.584, *p* = 0.002. Only the infected acquaintances (β = −0.169, *p* = 0.028) and health anxiety (β = 0.285, *p* = 0.003) had a significant unique contribution to the model.

*For general hygiene*, socio-demographic variables at Step 1 resulted in an insignificant model, explaining 6.9% of the variance in the outcome variable, *F*_change_(9,152) = 1.260, *p* = 0.263. Knowledge of COVID-19 at Step 2 was also not significant, explaining 0.7% of the variance, *F*_change_(1,151) = 1.160, *p* = 0.283. At Step 3, psychological factors added a significant 10.2%, *F*_change_(4,147) = 4.548, *p* = 0.002. The final model explained 10% of variance in general hygiene, as indexed by adjusted *R*^2^ statistic, *F*(14,147) = 2.278, *p* = 0.008. Education (β = −0.284, *p* < 0.001), perceived risk (β = 0.224, *p* = 0.015), and automatic thoughts (β = −0.195, *p* = 0.022) had a significant unique contribution to the model.

For *information seeking*, sociodemographic variables explained a nonsignificant 4% of variance in the outcome variable, *F*_change_(9,152) = 0.713, *p* = 0.697 and the addition of knowledge of COVID-19 at Step 2 was also nonsignificant, *F*_change_(1,151) = 0.001, *p* = 0.981, adding 0% to the model. However, psychological factors significantly predicted information seeking after controlling for sociodemographic variables, infected acquaintances and knowledge of COVID-19, *F*_change_(4,147) = 6.460, *p* < 0.001, adding 14.3% to the model. The final model explained 10.6% of variance in information seeking, *F*(14,147) = 2.367, *p* = 0.005. Only perceived risk (β = 0.297, *p* = 0.001) had a significant unique contribution to the model.

For *health behavior*, sociodemographic variables resulted in a nonsignificant model, *F*_change_(9,152) = 1.436, *p* = 0.177, explaining 7.8% of the variance in the outcome variable. The addition of knowledge of COVID-19 at Step 2 only added a nonsignificant 0.1%, *F*_change_(1,151) = 0.142, *p* = 0.707 and psychological factors at Step 3 a nonsignificant 4.4% to the model, *F*_change_(4,147) = 1.839, *p* = 0.124. The final model added only a nonsignificant 4% of variance in health behavior, *F*(14,147) = 1.474, *p* = 0.127.

### Results for Generation Z

At Step 1, socio-demographic variables contributed a non-significant 16.2% of variance. At Step 2, adding knowledge of COVID-19 added a non-significant 0.1% of variance, *F*_change_ (1,75) = 0.060, *p* = 0.807. At Step 3, however, the addition of psychological factors significantly added 19% of variance, *F*_change_(4,71) = 5.226, *p* = 0.001. The final model in predicting preventive behavior accounted for 23.4% of variance in preventive behavior scores, as indexed by adjusted *R*^2^ statistic, *F*(13,71) = 2.979, *p* = 0.002. The total effect size of the regression for Gen Z (*n* = 85) Cohen’s *f*^2^ = 0.295 was medium. Only psychological flexibility (β = 0.384, *p* = 0.001) made a unique significant contribution to the final model.

For *social distancing*, socio-demographic variables in Step 1 resulted in a nonsignificant model explaining 13% of variance [*F*_change_(8,76) = 1.417, *p* = 0.203] and by the addition of knowledge of COVID-19 at Step 2 explaining 0.5% of variance [*F*_change_(1,75) = 0.466, *p* = 0.497] or psychological factors at Step 3 explaining in addition 0.9% of variance [*F*_change_(4,71) = 0.184, *p* = 0.946], but the model still remained nonsignificant. The final model was nonsignificant, *F*(13,71) = 0.919, *p* = 0.538.

*For general hygiene*, socio-demographic variables in Step 1 resulted in a nonsignificant model explaining 10.6% of variance [*F*_change_(8,76) = 1.129, *p* = 0.354] and by the addition of knowledge of COVID-19 at Step 2 [*R*^2^_change_ < 0.001, *F*_change_(1,75) = 0.016, *p* = 0.898] or psychological factors at Step 3, explaining in addition 8.7% of variance [*F*_change_(4,71) = 1.904, *p* = 0.119], but the model still remained nonsignificant. The final model was nonsignificant *F*(13,71) = 1.306, *p* = 0.230.

For *information seeking*, socio-demographic variables in Step 1 explained a significant 19.1% of variance in the outcome variable, *F*_change_(8,76) = 2.239, *p* = 0.033. At Step 2, knowledge of COVID-19 did not have a significant contribution to the model, *R*^2^_change_ < 0.001, *F*_change_(1,75) = 0.032, *p* = 0.859. Psychological factors, however, at Step 3 added a significant 14.5% to the model, *F*_change_(4,71) = 3.886, *p* = 0.007. The final model explained 21.5% of the variance in information *seeking*, as indexed by *R*^2^ adjusted statistic, *F*(13,71) = 2.768, *p* = 0.003. Only marital status (other β = −0.207, *p* = 0.045) and psychological flexibility (β = 0.305, *p* = 0.007) contributed to the model.

For *health behavior*, socio-demographic variables in Step 1 explained nonsignificant 11.4% of variance in the outcome variable, at Step 2, *F*_change_(8,76) = 1.224, *p* = 0.297. Knowledge of COVID-19 did not have a significant contribution to the model (*R*^2^_change_ = 0.001, *F*_change_(1,75) = 0.070, *p* = 0.793). Psychological factors, however, at Step 3 added a significant 19.5% to the model, *F*_change_(4,71) = 5.010, *p* = 0.001. The final model explained 18.3% of the variance in health behavior, as indexed by *R*^2^ adjusted statistic, *F*(13,71) = 2.451, *p* = 0.008. Only psychological flexibility (β = 0.326, *p* = 0.005) had a contribution to the model.

## Discussion and Conclusion

The main aim of the current study was to identify psychological predictors of COVID-19 prevention behavior across different generations of women. To this day, knowledge of intergenerational characteristics concerned with specific COVID-19 prevention behavior domains is scarce in the literature, that is the reason why this explorative study is meant to provide new insight on this topic. The additional aim was to explore the psychological predictors in regard to the psychological variables linked to positive and negative information processing on distinct COVID-19 preventive domains (social distancing, general hygiene, information seeking, health behavior). Demographic variables and disease knowledge were previously investigated in the literature, but less was comprehensively written on the specific psychological variables chosen in this study (perceived risk, COVID-19 health anxiety, negative automatic thoughts, and psychological flexibility). One of the most researched psychological factor from the targeted variables was the perceived risk, which has a statistically significant association with prevention behavior (Mulia, 2019; Asmundson and Taylor, 2020; Dryhurst et al., 2020). Health anxiety, automatic negative thoughts and psychological flexibility were analyzed in the context of mental health issues and COVID-19, but they were not specifically linked to prevention behavior and this approach that we took, highlighted many new and diverse statistically significant associations.

Generational differences and effect sizes were calculated for all psychological variables. In case of perceived risk there were no generational differences identified. In case of COVID-19 knowledge, COVID-19 health anxiety and psychological flexibility generational differences were highlighted, but all effect sizes were small. In case of negative automatic thoughts generational differences were significant, but only between baby boomers and Gen Z was a moderated effect size present, the younger generation being characterized by a higher amount of negative automatic thoughts. A possible explanation can be found in the higher vulnerability for psychological distress and depression in this generation evidenced in the literature (Yavuzer and Karatas, 2017; Bethune, 2019).

Manifold research on COVID-19 evaluated *the role of demographic variables*, which often were associated with prevention behavior (Krewski et al., 2006; Ibuka et al., 2010; Olapegba et al., 2020; Singh et al., 2020; Zhong et al., 2020). The current study’s results suggested that the predictive power of demographic variables was low and statistically insignificant for prevention behavior pertaining to women of each generational group, when considering all the domains together. In a differentiated approach on COVID-19 prevention behavior domains (social distancing, general hygiene, information seeking, health behavior), significant predictors have appeared from the demographic variables, as well. From all the variables, the only one which showed a statistically significant role was the marital or the relationship status of the participants. The results are concordant with the literature, since marital status was previously identified to hold a significant role in health sustaining behavior, especially in individuals aged 40 or higher (Hilz and Wagner, 2018). The lack of a spouse increases the likelihood of health risk behavior and illness (Umberson, 1992; Norcross et al., 1996; Schone and Weinich, 1998; Kim et al., 2018) and this association seems to be present in the current predictive analysis as well, in case of information seeking and social distancing. Previous results regarding education highlight its positive predictive power (Singh et al., 2020), however, in the current research, education held a negative predictive role in relation to general hygiene only for Gen X and Y. This is partially in contradiction with the literature. A possible explanation can be found in unobserved variables, which could influence both health and education (Fonseca Benito and Zheng, 2011), but there is insufficient knowledge on this topic related to different generations.

The predictor role of *COVID-19 knowledge* on distinct prevention areas evinced some important diversity between generations. General hygiene and information seeking for baby boomers and general hygiene and social distancing for Generation X, positively associated with prevention behavior. However, when taking into account psychological predictors, knowledge of COVID-19 remained effective in predicting only preventive social distancing among Gen X women. Some findings suggest that knowledge is essential in prevention behavior (Smith, 2006; Janjua et al., 2007; Ilesanmi and Alele, 2016; Zegarra-Valdivia et al., 2020), but on the other hand knowledge was found insufficient for health behavior change and disease management (Duke et al., 2009; McAndrew et al., 2012). The findings of this study show a beneficial role of knowledge about COVID-19 in prevention especially in older individuals.

The hierarchical multiple regression, in which all sociodemographic variables and COVID-19 knowledge were statistically controlled for, showed patterns in predictive power on *total prevention behavior*, showing diversity among generations. The most important variable which made a statistically unique contribution, as a predictor of total prevention behavior in this case, was the perceived risk across generations. This finding is consistent with the literature (Krewski et al., 2006; Tandi et al., 2018; Morgan et al., 2019; Mulia, 2019). Furthermore, in regards to preventive behavior, COVID-19 health anxiety was positively associated and automatic negative thoughts were negatively associated predictors. Results are concordant with former findings (Khee et al., 2004; Krewski et al., 2006; Harper et al., 2020; Özdin and Özdin, 2020). Misinterpreting tendency on COVID-19 health information, which was assessed in the present study as a health anxiety tendency (Salkovskis et al., 2002), seemed to promote prevention behavior in Gen X. However, automatic negative thoughts seemed to undermine this prophylactic attitude. Surprisingly, in the case of Gen Z, only psychological flexibility had a significant unique contribution to the final model. Psychological flexibility was rarely analyzed in the context of prevention behavior in epidemiological events (Cheung and Mak, 2016; Dawson and Golijani-Moghaddam, 2020) and little is known about its role in Generation Z, until now.

To conduct a more profound explorative approach on the *distinct domains of COVID-19 prevention behavior*, they were examined separately in the prediction model which comprised the psychological factors and the results emphasized details in the dissimilarity between generations. In every instance, the sociodemographic variables and COVID-19 knowledge were statistically controlled and the predictive role of all assessed psychological factors was appraised separately for each distinct domain of prevention behavior: social distancing, general hygiene, information seeking, and health behavior. Distinct psychological constructs were positively associated with *social distancing* in different generations. With reference to baby boomers, risk perception had a relevant unique positive role. For Generation X, COVID-19 health anxiety, along with perceived risk increased preventive behavior, while with regard to Generation Y, only health anxiety was found to influence social distancing. The measured psychological characteristics were not relevant predictors for Generation Z. Similarly, to social distancing, the psychological predictors of *general hygiene* showed distinct patterns across generations. Baby boomers seemed to practice better general hygiene if COVID-19 perceived risk was higher. About Generation X, the perceived risk, the COVID-19 health anxiety proved to be statistically significant positive predictors, while automatic negative thoughts was a negative predictor. Results also evidenced that in relation to general hygiene, the perceived risk was positively associated and automatic negative thoughts was negatively associated. For Generation Z, the assessed psychological predictors did not account for variability in general hygiene. Perceived risk, COVID-19 health anxiety and unexpectedly, negative automatic thoughts increase *information seeking* for baby boomers. In the case of Gen X, both perceived risk and COVID-19 health anxiety were statistically significant individual positive predictors of information seeking. For Gen Y, the perceived risk and for Gen Z, the psychological flexibility accounted for the significant variability in seeking information. As to baby boomers, automatic negative thoughts (negative predictor) and the psychological flexibility (positive predictor) had a relevant predictive role in health behavior. The only generation where none of the assessed psychological constructs played a predictive role for *health behavior* was Gen Y. For Gen X, the perceived risk, the automatic thoughts, predicted negatively, while the psychological flexibility predicted positively health behavior. Psychological flexibility was relevant for Gen Z women.

*Risk perception*, which is linked to a general focus on COVID-19 threats, possibility of infection and danger of infection, was one of the most important predictors, for most generations, in many domains of prevention behavior. Gen Z was the only generation where risk perception was an irrelevant predictor variable. This result was only partly in line with the literature. As it was expected based on previous studies, perceived risk of COVID-19 played a positive role in social distancing, general hygiene and information seeking. Ibuka et al. (2010) and Krewski et al. (2006) found an association between perceived risk and the hygiene domain of prevention. The negative predictor role it plays for healthy behavior is in contradiction with other findings (e.g., Sheeran et al., 2014). One possible explanation can be that World Health Organization (2019, 2020a) insisted more upon the association of perceived risk, social distancing and hygiene guidelines with preventive behavior. Health behavior was rarely in the focus of COVID-19 prevention guidelines.

*Negative automatic thoughts* played mostly a negative role in prediction, especially in general hygiene (Gen X and Gen Y) and health behavior (baby boomers, Gen X, and Gen Y), which puts light on their disruptive role for preventive adjustment in epidemiological events. Despite the fact that this negative association was confirmed before (Khee et al., 2004), a new aspect was also evinced in the current study. Namely, information seeking, in baby boomers was predicted positively by negative automatic thoughts. This highlights the fact that negative information processing and focus on negative information might actually be worthy only in this area of COVID-19 preventive behavior, in case of older participants. This variable did not play a predictive role in any of the COVID-19 preventive behavior domains in case of Gen Z. Literature does not provide enough evidence on these aspects.

*COVID-19 health anxiety* predicted social distancing (Gen X), general hygiene (Gen X) and information seeking (baby boomers). In all cases a positive predictor role contoured. For the baby boomers, the result is in line with previous findings (Gerolimatos and Edelstein, 2012; American Psychiatric Association, 2017). The outcome found for Gen Z is noteworthy because in their case the COVID-19 health anxiety seemed to be irrelevant in case of every prevention area. Similarly, to negative automatic thoughts, new data is needed for the clarification related to generational diversity.

Although negative automatic thoughts and COVID-19 health anxiety, considered as predictors related to negative information processing, represent a risk for mental health (Hollon and Kendall, 1980; Salkovskis et al., 2002; Khee et al., 2004; Commodari, 2017; Harper et al., 2020), they can play at the same time a positive and a negative predictor role, dependent on the areas of individual preventive conduct during the pandemic. COVID-19 prevention behavior as conceptualized and operationalized in the current study, seems to be more influenced by negative information processing, concordantly with the literature (e.g., Baumeister et al., 2001). Negativity in thoughts (e.g., automatic negative thoughts) can work against COVID-19 protective behavior, undermining general hygiene and health actions, as found in the current study. There is a twofold role present in case of negative information related psychological variables.

*Psychological flexibility* is the only variable from this analysis which focuses on a positive reorganization of information (Maor et al., 2014). This factor was identified as significant for resilient coping during the COVID-19 lockdown and supported mental health (Pakenham et al., 2020; Smith et al., 2020). Interventions in psychological flexibility gained empirical support in addressing mental health problems caused by the COVID-19 pandemic (Landi et al., 2020; Polizzi et al., 2020; Presti et al., 2020). Better strategies of personal adjustment were facilitated by psychological flexibility and lowered the chance of mental health problems (Dawson and Golijani-Moghaddam, 2020). Considering prevention behavior domains, the existing results showed a significant role of psychological flexibility especially in health-related behaviors (exercise, sleep, and diet) in three generations (baby boomers, Gen X, and Z). This result confirms previous findings that revealed the importance of psychological flexibility in prevention behavior (Cheung and Mak, 2016). The current research provides additional support for generational differences, because in respect of psychological flexibility, it was identified as a positive predictor of information seeking only for Gen Z. This finding was not underlined before in the literature.

Taking all psychological predictors into account, it can be concluded that the role of different factors differs across domains of prevention health behavior. Inside the domains of prevention with a medical priority (social distancing and hygiene) negative information processing factors, whilst in other areas (information seeking and health behavior) positive information related to psychological flexibility seemed to count more. The impact of generational identity on health prevention behavior during the current COVID-19 pandemic seems relevant. Generational cohorts may share important characteristics (Park and Gursoy, 2012), which can influence their reactions to the COVID-19 pandemic. Also, the coexistence of negative and positive information processing may have its beneficial role in certain areas of prevention. The focus on possible bad consequences was found to be important for primary prevention of COVID-19 (social distancing and general hygiene), however the negative automatic thoughts can undermine prevention. Psychological flexibility as a protective factor for mental health is important in information seeking and in health behavior.

### Limitations and Future Direction

Beyond the new findings that highlight the diversity in prevention behavior predictors across generations, several limitations were identified. First, the psychological factors were assessed by self-reported measures, which potentially can induce bias in the interpretation of the results. Second, the cross-sectional, one-time measurement design cannot assess information on dynamics and alterations in COVID-19 prevention behavior. The newly developed instrument on COVID-19 prevention behavior had two subscales with a moderated reliability (health behavior and information seeking), which hold certain interpretational risks and there is a need for further studies. Moreover, the recruitment of the sample was made online, by convenience sampling method, without any control or prior assessment of psychological well-being. Although the total sample was adequate for analyses, the sample sizes of the four generational cohorts was not suitable for detecting small effect sizes. The sample was recruited from several European countries with different policy measures imposed by the government. These and other possible cultural characteristics could have influenced some aspects of preventive behavior. The cultural moderation hypothesis should be tested in a cross-cultural research design. The results do not allow to infer any causality; thus future research could explore the mechanisms behind the generational diversity of COVID-19 preventive behavior predictors. There is a lack of studies that focused on generational differences in epidemiological events. More details are needed in case of preventive behavior of Gen Z, particularly on the distinctive data we have found (e.g., on the role of psychological flexibility). The direction of relationship between predictive behavior and other variables, like education, negative automatic thoughts, and COVID-19 health anxiety needs further elucidation, while it presents many contradictions with the literature or there is a lack of previous studies which can help possible explanations. The outcome found for Gen Z on of perceived risk, COVID-19 health anxiety and negative automatic thoughts needs further investigation. The positive role of psychological flexibility on health behavior shows across many generations (baby boomer, Gen X, and Gen Z), and this variable was identified as a positive predictor of information seeking only for Gen Z, which needs further clarification. The presence of interpretational biases (e.g., positive and negative illusions), flexible adaptation to the health-related aspects of the pandemic and self-related conceptualization of these undesired circumstances might be potential underlying mechanisms of generational differences (e.g., negative automatic thoughts in baby boomer vs. Gen Z). Further research is needed for clearer conclusions and possible explanations.

## Data Availability Statement

The dataset that support the findings of this study are openly available in Figshare at https://doi.org/10.6084/m9.figshare.13379696.v1.

## Ethics Statement

The studies involving human participants were reviewed and approved by the Ethics Committee of the University of Babeş-Bolyai (RO). The participants provided their written informed consent to participate in this study.

## Author Contributions

All authors have contributed equally to conception and design of the study, statistical analysis, writing, manuscript revision, and approved the submitted version.

## Conflict of Interest

The authors declare that the research was conducted in the absence of any commercial or financial relationships that could be construed as a potential conflict of interest.
